# Longitudinal study of cardiometabolic risk from early adolescence to early adulthood in an ethnically diverse cohort

**DOI:** 10.1136/bmjopen-2016-013221

**Published:** 2016-12-15

**Authors:** Seeromanie Harding, Maria João Silva, Oarabile R Molaodi, Zinat E Enayat, Aidan Cassidy, Alexis Karamanos, Ursula M Read, J Kennedy Cruickshank

**Affiliations:** 1 Cardiovascular Medicine & Social Epidemiology Group, Division of Diabetes & Nutritional Sciences, King's College London, London, UK; 2 MRC/CSO Social and Public Health Sciences Unit, Institute of Health and Wellbeing, University of Glasgow, Glasgow, UK; 3 National Hospital for Neurology and Neurosurgery, University College London Hospitals, London, UK; 4 Department of Epidemiology and Health, ESRC International Centre for Lifecourse Studies in Society and Health, University College London, London, UK; 5 CERMES3 (Centre de Recherche Médecine, Sciences, Santé, Santé Mentale et Société), Université Paris Descartes, EHESS, CNRS UMR 8211, INSERM U988, Paris, France

**Keywords:** ethnicity, cohort, growth, later CVD risk, overweight, adolescence

## Abstract

**Objective:**

To examine influences of adiposity from early adolescence to early 20s on cardiovascular disease (CVD) risk in the multiethnic Determinants of young Adult Social well-being and Health (DASH) longitudinal study.

**Methods:**

In 2002–2003, 6643 11–13-year-olds from 51 London schools participated at baseline, and 4785 were seen again at 14–16 years. Recently, 665 (97% of invited) participated in pilot follow-up at 21–23 years, with biological and psychosocial measures and blood biomarkers (only at 21–23 years). Regression models examined interplay between ethnicity, adiposity and CVD.

**Results:**

At 21–23 years, ∼30–40% were overweight. About half of the sample had completed a degree with little ethnic variation despite more socioeconomic disadvantage in adolescence among ethnic minorities. Regardless of ethnicity, overweight increased more steeply between 14–16 years and 21–23 years than between 11–13 years and 14–16 years. More overweight among Black Caribbean and Black African females, lower systolic blood pressure (sBP) among Indian females and Pakistani/Bangladeshi males compared with White UK peers, persisted from 11–13 years. At 21–23 years, glycated haemoglobin (HbA1c) was higher among Black Caribbean females, total cholesterol higher and high-density lipoprotein (HDL) cholesterol lower among Pakistani/Bangladeshis. Overweight was associated with a ∼+2 mm Hg rise in sBP between 11–13 years and 21–23 years. Adiposity measures at 11–13 years were related to allostatic load (a cluster of several risk markers), HbA1c and HDL cholesterol at 21–23 years. Ethnic patterns in CVD biomarkers remained after adjustments.

**Conclusions:**

Adolescent adiposity posed significant risks at 21–23 years, a period in the lifespan generally ignored in cardiovascular studies, when ethnic/gender variations in CVD are already apparent.

**Strengths and limitations of this study**

- Determinants of young Adult Social well-being and Health (DASH) is one of the few cohorts with an ethnically diverse composition in their 20s, a time when physical health is at its peak, yet when early signs of disease begin to appear.
- DASH has high retention rates and provides a comprehensive picture of the ethnic patterning of growth from early adolescence.
- It is the first UK study, as yet from a pilot follow-up, to report a prospective association between adiposity measures and cardiovascular disease risk from early adolescence to early adulthood in an ethnically diverse cohort.
- The lack of data before age 11 years, blood biomarkers only at 21–23 years and pilot sample size at 21–23 years are limitations.

## Introduction

The UK has a rapidly growing ethnically diverse population. In the 2011 Census, the non-White British population in the UK was 20% of the population and 55% in London. The public health implications are significant given the well-documented ethnic disparities in cardiometabolic disease. People of South Asian or Black African descent have, respectively, fourfold and threefold elevated risks of diabetes compared with White Europeans.^1^ ^2^ Risks of hypertension and stroke are also greater in these groups, but while South Asians also experience higher rates of coronary disease, people of Black African origin are protected. There is increasing evidence that ethnic differences in risk factors such as blood pressure (BP) emerge in childhood with greater metabolic sensitivity to adiposity than Whites^3^ including from infancy to 3-year-old.^4–6^ Black African and Black Caribbean girls are more likely to be overweight, and Indian girls to have larger waists (adjusted for height) in childhood compared with their White UK counterparts. Ethnic differences in cardiovascular (CV) health also persist after adjustment for measures for socioeconomic circumstances (SEC). However, considerable debate continues as to whether the association is causal, particularly as most studies are cross-sectional.

Obesity, and its metabolic and haemodynamic consequences, underpins a significant proportion of adult ill health. The Foresight Obesity project forecasted that by 2050, 60% of men and 50% of women will be obese, with growing ethnic disparities projected. The impact of obesity acquired during rapid somatic growth on cardiometabolic disease is unclear, and the tracking of obesity from adolescence into early adulthood may account better for ethnic differences in disease. In the pilot follow-up of our Determinants of young Adult Social well-being and Health (DASH) study, we used an initial panel of biomarkers, traditional and novel, neither often measured at this age. Lipid profiles and glycosylated haemoglobin (HbA1c by which type 2 diabetes can now be defined) were included. Inflammation in adipose tissue is likely to play a key role in vascular damage preceding overt diabetes.^7^ ^8^ Allostatic load (AL), a cluster of several risk markers, is proposed as a biological link accounting for Black–White/socioeconomic disparities in morbidity and mortality in the USA, representing the cumulative dysregulation of biological systems. It is patterned by social determinants (eg, ethnicity, education) and prospectively predicts mortality as well as cognitive and physical decline.^9^ ^10^ Studies of ethnic differences in AL are mainly US-based, where Black–White disparity emerges by 35 years, 5 years earlier than for the UK Black Caribbeans.^11^ We recently showed that by age 30 years, most ethnic minority groups had higher AL than their general population peers, and that living in deprived neighbourhoods was associated with greater biological wear and tear, independent of individual SEC.^11^

There are few cohorts with an ethnically diverse composition in early adulthood, a time when physical health is at its peak, yet when early signs of disease begin to appear. Studies that tracked growth from childhood and collected biomarkers and vascular measures are mainly of White Europeans with few in Europe varying in ethnic make-up.^12^ ^13^ The DASH study, established in 2002, has followed health and social exposures of over 6000 young Londoners (UK), including 80% ethnic minorities, over the last 12 years. A pilot follow-up study of the cohort, now in their early 20s, was recently completed. Here, we examine pilot-study phenotypes for potential ethnic patterns from early adolescence to early 20s in (1) body size and BP trajectories; (2) the influence of body size on BP and on vascular biomarkers (HbA1c, total (TC) and high-density lipoprotein (HDL) cholesterol and AL) and (3) whether gender and SEC influenced ethnic variations in risks.

## Materials and methods

Details of the DASH study can be found elsewhere.^14^ In 2002–2003, a total of 6643 students, aged 11–13 years, from 51 secondary schools in 10 London boroughs, took part at baseline. In 2005–2006, 4785 (88% of children in 49 schools, 72% of the initial cohort) participated in the first follow-up aged 14–16 years. A 10% subsample (N=665, 97% participation rate) took part in the pilot follow-up completed in March 2014. The subsample consisted of 107 White UK, 102 Black Caribbean, 132 Black African, 99 Indian, 111 Bangladeshi or Pakistani and 115 other (mainly mixed) ethnicities. We first tried to locate the sample and 81% (5414 of 6643) of the cohort was traced through friendship networks, social media, and community campaigns. We then randomly selected 100 (50 per gender) in each in ethnic group, and pragmatically attempted, by repeated random selection, to ensure representation across the London boroughs, schools and SEC at 11–13 years.

Written informed consent was obtained from all participants. Ethnicity was self-reported, checked against reported parental ethnicity and grandparents' country of birth. Bangladeshis and Pakistanis were combined due to small sample sizes.

### Body size, biological and social measures

Field staff were trained for 1 week prior to the start of fieldwork, and were recertified at 6-monthly intervals. Equipment was calibrated regularly by the field supervisors. During adolescence, assessments were conducted in schools and at 21–23 years, in community locations (eg, local general practitioner's surgeries, local community pharmacies, King’s College London).

Body Mass Index (BMI) can underestimate body fat among South Asians and overestimate among African-origin populations.^15^ In the absence of bioelectrical impedance at all ages, we used waist to height ratio (WHtR) and overweight/obesity status (referred to as overweight hereafter). Height was measured using portable stadiometers, to the nearest 0.1 cm and weight using Salter electronic scales, to the nearest 0.1 kg. In adolescence, participants were classified as normal weight, overweight or obese using the International Obesity Task Force, based on the 1990 British growth reference curves.^16^ At 21–23 years, overweight was classified as a BMI of 25–29.9 kg/m^2^, obese a BMI of ≥30 kg/m^2^. Waist circumference (cm) was measured midway between the 10th rib and the top of the iliac crest, and 0.5 cm subtracted to correct for measurement over T-shirt or vest. The mean of two duplicate measures was derived for the WHtR.

BP was measured using validated OMRON M5–1 instruments and appropriately sized cuffs, after sitting quietly for a timed 5 min, with >1 min between three subsequent readings. The mean of the second and third readings was used in analysis. A 25 mL blood sample was taken by venepuncture at 21–23 years. We examined the following seven biomarkers: systolic blood pressure (sBP), diastolic blood pressure (dBP), WHtR, BMI, TC, HDL cholesterol and HbA1c. These biomarkers were used to create AL, based on the highest risk quartile values of the sample distribution.^17^ A biomarker reading beyond the threshold (<25th centile for HDL cholesterol, >75th centile for all other biomarkers) was assigned one point, corresponding to thresholds of: sBP 121/dBP 77 mm Hg; BMI 26.8 kg/m^2^; WHtR male 0.89, female 0.86; TC 4.8 mmol/L; HDL cholesterol 1.8 mmol/L; HbA1c 37.0 mmol/mol. AL score was derived by summing the points.

A self-complete questionnaire measured smoking, SEC (as family affluence and parental employment in adolescence, own education and employment at 21–23 years).

### Statistical analysis

Continuous variables (sBP, dBP, WHtR, BMI, TC, HDL cholesterol and HbA1c) did not require transformation as the Shapiro-Wilk test indicated normal distributions. All models were gender-stratified. We first examined potential effects of age (used as single years) and ethnicity, adjusted for each other, on outcomes. Ethnic-specific effects were tested using interaction terms ethnicity × age and ethnicity × adiposity. Measures of adiposity (WHtR, overweight) were added to the core model with age and ethnicity, using separate models for each adiposity measure. Models were gender-stratified. Social correlates (SEC, smoking) were added to the models only if they were statistically significant in univariable analyses and where the log likelihood ratios indicated better fitted models.

Mixed-effects models examined longitudinal outcomes for sBP and adiposity measures (WHtR—linear models, overweight/obese vs normal weight—logistic models). Linear regression models were used for HbA1c, HDL, TC and AL. Two sets of models were run. The first set was with adiposity measures at 11–13 years to examine whether, at this critical period of adolescence, there was an association with biomarkers at 21–23 years. The second investigated the influence of change in adiposity measures between 11–13 years and 21–23 years. Stata V.13 was used for all analyses. Missing data among continuous variables were <5% for BP and anthropometric variables (N=664 for mixed-effects models with BP as outcome) and 24% for blood biomarkers (N=502 for linear regression models). Missing data for categorical covariates were coded and included in the analyses. Statistical significance was considered at p<0.05.

## Results

Table 1 shows distributions by social correlates, ethnicity and age. Ethnic minorities were generally less likely to smoke, Black Africans least. Completion of a degree did not vary significantly, despite more socioeconomic disadvantage in adolescence among the ethnic minority groups.

**Table 1:** Descriptive profile of longitudinal pilot follow-up sample: percentage (95% CI) by ethnicity

	White UK (N=107)	Black African (N=132)	Black Caribbean (N=102)	Indian (N=98)	Pakistani Bangladeshi (N=111)	Others (N=115)	All (N=665)
*Socioeconomic circumstances at 11–13 years*							
Parental unemployment							
≥1 employed	89.7 (82.3 to 94.2)	76.5 (68.5 to 83.0)	77.4 (68.3 to 84.6)	75.6 (66.0 to 83.1)	57.7 (48.2 to 66.5)	80.0 (71.6 to 86.4)	76.1 (72.7 to 79.2)
None employed	5.6 (2.5 to 12.0)	15.1 (10.0 to 22.4)	15.7 (9.8 to 24.1)	12.2 (7.1 to 20.4)	30.6 (22.7 to 39.9)	13.9 (8.7 to 21.6)	15.6 (13.1 to 18.6)
Not stated	4.7 (1.9 to 10.8)	8.3 (4.7 to 14.4)	6.9 (3.3 to 13.8)	12.2 (7.1 to 20.4)	11.7 (6.9 to 19.2)	6.1 (2.9 to 12.3)	8.3 (6.4 to 10.6)
Family Affluence Scale*							
≥3	60.7 (51.1 to 69.6)	47.7 (39.3 to 56.3)	50.0 (40.3 to 59.6)	50.0 (40.2 to 59.8)	43.2 (34.3 to 52.6)	60.0 (50.7 to 68.6)	51.9 (48.1 to 55.7)
1–2	29.0 (21.1 to 38.3)	27.3 (20.3 to 35.5)	32.4 (23.9 to 42.1)	30.6 (22.2 to 40.5)	36.0 (27.6 to 45.4)	27.8 (20.4 to 36.7)	30.4 (27.0 to 34.0)
0	1.0 (1.2 to 6.4)	1.5 (0.4 to 5.9)	7.8 (3.9 to 15.0)	2.0 (0.5 to 78.9)	3.6 (1.3 to 9.3)	1.7 (0.4 to 6.7)	2.8 (1.8 to 4.4)
Not stated	9.3 (5.1 to 16.6)	23.5 (17.0 to 31.5)	9.8 (5.3 to 17.3)	10.4 (6.0 to 17.5)	17.2 (11.2 to 25.3)	10.4 (6.0 to 17.5)	14.9 (12.4 to 17.8)
*Socioeconomic circumstances at 14–16 years*							
Parental unemployment							
≥1 employed	86.0 (78.0 to 91.4)	81.8 (74.2 to 87.5)	79.4 (70.4 to 86.2)	84.7 (76.1 to 90.6)	57.7 (48.2 to 66.5)	35.7 (27.4 to 44.8)	70.5 (67.0 to 73.9)
None employed	0.9 (0.1 to 6.4)	12.1 (7.5 to 18.9)	13.7 (8.3 to 21.9)	7.1 (3.4 to 14.3)	30.6 (22.7 to 39.9)	4.3 (1.8 to 10.1)	11.6 (9.3 to 14.2)
Not stated	13.1 (7.9 to 20.9)	6.1 (3.0 to 11.7)	6.9 (3.3 to 13.8)	8.2 (4.1 to 15.5)	11.7 (6.9 to 19.2)	60.0 (50.7 to 68.6)	17.9 (15.1 to 21.0)
Family Affluence Scale*							
≥3	60.7 (51.1 to 70.0)	47.7 (39.3 to 56.3)	50.0 (40.3 to 59.6)	50.0 (40.2 to 59.8)	43.2 (34.3 to 52.6)	60.0 (50.7 to 68.6)	61.6 (57.9 to 65.3)
1–2	29.0 (21.1 to 38.3)	27.3 (20.3 to 35.5)	32.3 (23.9 to 42.1)	30.6 (22.2 to 40.5)	36.0 (27.6 to 45.4)	27.8 (20.4 to 36.7)	27.5 (24.2 to 31.0)
0	1.0 (0.1 to 6.4)	1.5 (0.4 to 5.9)	7.8 (3.9 to 15.0)	2.1 (0.5 to 7.9)	3.6 (1.3 to 9.3)	1.7 (0.4 to 6.7)	1.7 (0.9 to 3.0)
Not stated	9.3 (5.1 to 15.6)	23.5 (17.0 to 31.5)	9.8 (5.3 to 17.3)	17.3 (11.0 to 26.2)	17.2 (11.2 to 25.3)	10.4 (6.0 to 17.5)	9.2 (7.2 to 11.6)
*Socioeconomic circumstances at 21–23 years*							
Own employment							
Yes	49.5 (40.1 to 59.0)	45.5 (37.1 to 54.0)	53.9 (44.1 to 63.4)	48.0 (38.2 to 57.9)	39.7 (30.9 to 40.1)	48.7 (39.6 to 57.8)	47.4 (42.6 to 51.2)
No	42.1 (33.0 to 51.6)	37.1 (29.3 to 45.7)	33.3 (24.8 to 43.1)	37.7 (28.7 to 47.8)	42.3 (33.5 to 51.7)	37.8 (29.0 to 46.6)	38.3 (34.7 to 42.1)
Not stated	8.4 (4.4 to 15.4)	17.4 (11.8 to 24.9)	12.8 (7.5 to 20.8)	14.3 (8.6 to 22.8)	18.0 (11.9 to 26.3)	13.9 (8.7 to 2.6)	14.3 (11.8 to 17.2)
Education							
Not educated to a degree level	51.4 (41.9 to 60.8)	33.3 (25.8 to 41.8)	58.8 (49.0 to 68.0)	32.6 (24.1 to 42.6)	38.7 (30.1 to 48.1)	46.1 (37.1 to 55.3)	43.2 (39.4 to 47.0)
Educated to a degree level	40.2 (31.3 to 49.8)	57.6 (49.0 to 65.8)	34.3 (25.7 to 44.1)	57.1 (47.1 to 66.6)	48.6 (39.4 to 57.9)	43.5 (34.7 to 52.7)	47.2 (43.4 to 51.0)
Not stated	8.4 (4.4 to 15.4)	9.1 (5.2 to 15.4)	6.9 (3.3 to 13.8)	10.2 (5.5 to 18.0)	12.6 (7.6 to 20.2)	10.4 (6.0 to 17.5)	9.6 (7.6 to 12.1)
Smoking							
11–13 years	2.8 (0.9 to 8.4)	0	4.9 (2.0 to 11.3)	0	0.9 (0.1 to 6.2)	0.9 (1.2 to 6.2)	1.5 (0.8 to 2.8)
Not stated	8.4 (4.4 to 15.4)	29.5 (22.3 to 37.9)	15.7 (9.8 to 24.1)	17.3 (11.0 to 26.2)	18.0 (11.9 to 26.3)	19.1 (12.9 to 27.4)	18.5 (15.7 to 21.6)
14–16 year	15.9 (10.1 to 24.1)	8.8 (4.6 to 16.1)	2.3 (0.7 to 6.8)	3.1 (1.0 to 9.1)	2.7 (0.1 to 8.1)	12.2 (7.3 to 19.6)	7.4 (5.6 to 9.6)
Not stated	5.6 (2.5 to 12.0)	4.9 (2.0 to 11.3)	6.8 (3.6 to 12.6)	7.1 (3.4 to 14.3)	6.3 (3.0 to 12.7)	10.4 (6.0 to 17.5)	6.9 (5.2 to 9.1)
21–23 years	46.7 (37.4 to 56.2)	10.6 (6.4 to 17.1)	20.6 (13.8 to 29.6)	23.5 (16.1 to 32.9)	24.3 (17.2 to 33.2)	23.5 (16.6 to 32.1)	24.4 (21.2 to 27.8)
Not stated	21.5 (14.7 to 30.3)	53.0 (44.5 to 61.4)	41.2 (32.0 to 51.0)	46.9 (37.2 to 56.9)	47.7 (38.6 to 57.1)	38.3 (29.8 to 47.5)	41.8 (38.1 to 45.6)

*Family affluence was derived from the number of cars or vans, computers and holidays abroad each year.

### Ethnic patterns in body size and BP trajectories from early adolescence to early 20s

**Figure 1:**
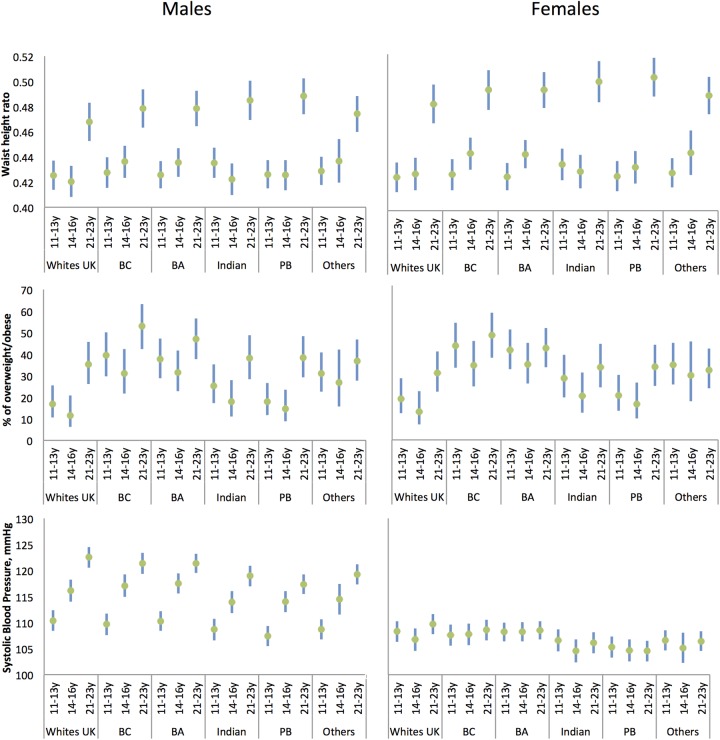
Waist to height ratio, per cent of overweight/obese and systolic blood pressure by age and ethnicity for males and females: means/percentage and 95% CIs adjusted for gender and ethnicity. BA, Black African; BC, Black Caribbean; PB, Pakistani/Bangladeshi.

Figure 1 and online supplementary table S1 show CV markers by ethnicity and gender.

10.1136/bmjopen-2016-013221.supp1 supplementary data

At 11–13 years, Black Caribbeans and Black Africans were 2–3 cm taller, Indians ∼5 cm shorter and Pakistanis/Bangladeshis ∼2 cm shorter than White UK. At 14–16 and 21–23 years, however, the White UK–ethnic minority difference was evident only for South Asians, who remained shorter (see online supplementary table S1).

Figure 1 and online supplementary table S1 show that from adolescence there was a pattern of more overweight/obesity for Black Caribbeans and Black Africans than their White UK peers. In every ethnic group, for males and females, there was a marked increase in WHtR between 14–16 years and 21–23 years, significantly more for Pakistanis/Bangladeshis than for White UK. sBP rose more in males than females. While sBP rose more in adolescence between 14–16 years and 21–23 years for Black Caribbean and Black African males, the opposite was observed for White UK.

At 21–23 years, compared with their White UK peers, mean HbA1c was higher among Black Caribbean females, mean TC higher among Pakistani/Bangladeshi males and mean HDL cholesterol lower among Indian and Pakistani/Bangladeshi males (see online supplementary table S1).

### The influence of body size on BP and biomarkers

Table 2 shows the influence of longitudinal measures of WHtR and overweight from 11–13 years to 21–23 years on sBP by gender. These indices were independent longitudinal correlates of sBP (and dBP—see online supplementary table S2). For example, between 11–13 years and 21–23 years overweight among females was associated with an average rise of +2.89 mm Hg (1.43–4.34) in sBP, and +3.09 (1.70–4.48) for dBP (see online supplementary table S2) compared with normal weight. The addition of SEC or smoking did not substantively change the results in the models for BP. Despite more overweight among females than males from adolescence, in gender-adjusted models, mean sBP was lower (−7.95 mm Hg, 95% CI −9.10 to −6.79). In these models with additional adjustments, the pattern of generally lower sBP for many ethnic minority groups was observed before and after adjustment for the different adiposity indices.

**Table 2:** Systolic blood pressure from adolescence to early adulthood for males and females: association with longitudinal measures of adiposity*

	Waist to height ratio			Overweight status		
	Coef	95% CI	p Value	Coef	95% CI	p Value
Males						
Waist to height ratio	25.94	13.72 to 38.16	<0.001	–	–	–
Overweight (normal weight—Ref)						
Overweight/obese	–	–	–	1.60	−0.25 to 3.46	0.091
Age (11–13 years—Ref) (years)						
14–16	6.73	5.39 to 8.08	<0.001	6.62	5.28 to 7.96	<0.001
21–23	8.98	7.52 to 10.44	<0.001	10.57	9.15 to 12.0	<0.001
Ethnicity (White UK—Ref)						
Black Caribbean	−1.08	−4.30 to 2.14	0.512	−1.44	−4.73 to 1.85	0.39
Black African	−0.97	−4.03 to 2.09	0.532	−1.25	−4.38 to 1.88	0.434
Indian	−3.16	−6.30 to −0.02	0.048	−3.14	−6.34 to 0.06	0.055
Pakistani/Bangladeshi	−5.58	−8.60 to −2.56	<0.001	−5.32	−8.41 to −2.23	0.001
Others	−4.26	−7.38 to −1.14	<0.001	−4.26	−7.45 to −1.07	0.009
Employment						
No	0.43	−1.17 to 2.04	0.6	0.27	−13.1 to 1.85	0.741
Not stated	1.24	−1.04 to 3.54	0.286	0.72	−1.53 to 2.97	0.533
Smoking						
Yes	0.89	−0.64 to 2.42	0.253	0.92	−0.60 to 2.44	0.238
Not stated	2.58	0.82 to 4.33	0.004	2.73	0.99 to 4.46	0.002
Females						
Waist to height ratio	30.18	20.98 to 39.37	<0.001	–	–	–
Overweight (normal weight—Ref)						
Overweight/obese	–	–	–	2.89	1.43 to 4.34	<0.001
Age (11–13 years—Ref) (years)						
14–16	−0.94	−2.05 to 16.81	0.096	−0.52	−1.63 to 0.60	0.569
21–23	−1.66	−2.93 to −0.39	0.082	1.17	−0.04 to 2.38	0.058
Ethnicity (White UK—Ref)						
Black Caribbean	−0.38	−2.82 to 2.06	0.758	0.06	−2.42 to 2.54	0.961
Black African	0.24	−2.06 to 2.55	0.836	0.45	−1.88 to 2.79	0.703
Indian	−2.63	−5.19 to −0.06	0.045	−2.19	−4.79 to 0.41	0.099
Pakistani/Bangladeshi	−2.31	−4.83 to 0.20	0.071	−1.93	−4.5 to 0.63	0.139
Others	−0.84	−3.29 to 1.61	0.502	−0.72	−3.22 to 1.77	0.569
Employment						
No	−0.39	−1.64 to 0.86	0.543	−0.40	−1.65 to 0.65	0.527
Not stated	1.59	−0.64 to 3.82	0.162	1.41	−0.77 to 3.59	0.204
Smoking						
Yes	0.54	−0.76 to 1.85	0.418	0.66	−0.64 to 1.97	0.317
Not stated	0.46	−0.91 to 1.84	0.508	0.22	−1.14 to 1.59	0.755

*Mixed-effects linear regression model for systolic blood pressure: regression coefficients adjusted for age, ethnicity, waist to height ratio or overweight (separate models), parental/own employment and currently smoking.

Tables 3 (males) and 4 (females) show the influence of WHtR and overweight status at 11–13 years on per unit change in vascular biomarkers at 21–23 years, by gender. Adiposity at 11–13 years was related to three (AL, HbA1c, HDL cholesterol) of four biomarkers. The corresponding results for change in adiposity are in online supplementary table S3 and online supplementary table S4. Increasing WHtR was associated with a decrease in HDL cholesterol and an increase in TC, and overweight at 11–13 years and 21–23 years with a decrease in HDL cholesterol (see online supplementary table S3). The unadjusted ethnic and gender patterns largely remained after adjustment for adiposity. In all of these models, there were no discernible ethnic-specific effects as tested by the interactions ethnicity × age, and ethnicity × adiposity measure.

**Table 3:** Males: the influence of adiposity at 11–13 years on biomarkers at 21–23 years*

	Allostatic load						HbA1c					
	Waist to height ratio			Overweight status			Waist to height ratio			Overweight status		
	Coef	95% CI	p Value	Coef	95% CI	p Value	Coef	95% CI	p Value	Coef	95% CI	p Value
Waist to height ratio, 11–13 years	7.69	4.27 to 11.11	<0.001	–	–	–	3.31	−5.33 to 11.92	0.450	–	–	–
Overweight, 11–13 years (normal weight—Ref)												
Overweight/obese	–	–	–	0.87	0.44 to 1.31	<0.001	–	–	–	1.06	−0.03 to 2.15	0.057
Ethnicity (White UK—Ref)												
Black Caribbean	−0.07	−0.77 to 0.63	0.843	−0.32	−1.02 to 0.38	0.365	0.93	−0.84 to 2.70	0.301	0.61	−1.14 to 2.35	0.493
Black African	−0.38	−1.06 to 0.30	0.272	−0.74	−1.42 to −0.06	0.033	1.04	−0.66 to 2.74	0.228	0.81	−0.87 to 2.50	0.344
Indian	−0.26	−0.92 to 0.41	0.449	−0.30	−0.97 to 0.37	0.383	1.35	−0.31 to 3.03	0.111	1.35	−0.32 to 3.02	0.113
Pakistani/Bangladeshi	−0.07	−0.72 to 0.59	0.835	−0.11	−0.77 to 0.55	0.751	0.71	−0.90 to 2.33	0.383	0.58	−1.02 to 2.18	0.478
Others	−0.35	−0.99 to 0.30	0.289	−0.50	−1.15 to 0.15	0.133	0.10	−1.53 to 1.71	0.848	−0.07	−1.70 to 1.55	0.929
	HDL cholesterol						Total cholesterol					
	Waist to height ratio			Overweight status			Waist to height ratio			Overweight status		
	Coef	95% CI	p Value	Coef	95% CI	p Value	Coef	95% CI	p Value	Coef	95% CI	p Value
Waist to height ratio, 11–13 years	0.2	−0.5 to 0.23	0.568	–	–	–	0.96	−0.82 to 2.73	0.289	–	–	–
Overweight, 11–13 years (normal weight—Ref)												
Overweight/obese	–	–	–	−0.03	−0.12 to 0.06	0.529	–	–	–	−0.01	−0.23 to 0.22	0.968
Ethnicity (White UK—Ref)												
Black Caribbean	0.09	−0.05 to 0.23	0.207	0.10	−0.04 to 0.24	0.165	−0.02	−0.38 to 0.34	0.914	−0.05	−0.40 to 0.31	0.788
Black African	0.06	−0.13 to 0.14	0.932	0.01	−0.13 to 0.14	0.965	−0.24	−0.59 to 0.11	0.179	−0.27	−0.61 to 0.08	0.132
Indian	−0.13	−0.27 to 0.02	0.054	−0.15	−0.29 to −0.01	0.03	0.09	−0.26 to 0.43	0.609	0.10	−0.24 to 0.45	0.548
Pakistani/Bangladeshi	−0.16	−0.29 to −0.03	0.018	−0.18	−0.30 to −0.04	0.01	0.35	−0.02 to 0.68	0.040	0.35	0.02 to 0.68	0.036
Others	0.02	−0.11 to 0.16	0.568	0.02	−0.12 to 0.16	0.74	0.03	−0.30 to 0.36	0.860	0.02	−0.31 to 0.36	0.883

*Linear regression model with regression coefficients adjusted for ethnicity and waist to height ratio or overweight status at 11–13 years. Allostatic load score derived at 21–23 years using high-risk thresholds defined as below the 25th centile for high-density lipoprotein (HDL) cholesterol, and above the 75th centile for all other biomarkers. The thresholds were: systolic, 121.0 mm Hg; diastolic, 77 mm Hg; BMI, 26.8 kg/m2; waist to height ratio male, 0.89, and female, 0.86; total cholesterol, 4.8 mmol/L; HDL cholesterol, 1.8 mmol/L; glycated haemoglobin (HbA1c), 37.0 mmol/mol.

**Table 4:** Females: the influence of adiposity at 11–13 years on biomarkers at 21–23 years in the DASH study*

Covariates	Allostatic load						HbA1c					
	Waist to height ratio			Overweight status			Waist to height ratio			Overweight status		
	Coef	95% CI	p Value	Coef	95% CI	p Value	Coef	95% CI	p Value	Coef	95% CI	p Value
Waist to height ratio, 11–13 years	11.36	7.76 to 17.95	<0.001	–	–	–	22.12	8.65 to 33.59	0.007	–	–	–
Overweight, 11–13 years (normal weight—Ref)												
Overweight/obese	–	–	–	1.23	0.80 to 1.67	<0.001	–	–	–	1.68	0.16 to 3.18	0.029
Ethnicity (White UK—Ref)												
Black Caribbean	0.22	−0.42 to 0.85	0.5	0.04	−0.61 to 0.69	0.903	2.65	0.32 to 4.98	0.026	2.51	0.16 to 4.86	0.037
Black African	−0.11	−0.75 to 0.52	0.723	−0.25	−0.89 to 0.39	0.439	0.4	−1.84 to 2.64	0.725	0.39	−1.87 to 2.66	0.732
Indian	−0.2	−0.88 to 0.48	0.56	−0.06	−0.74 to 0.63	0.871	−0.04	−2.54 to 2.46	0.973	0.19	−2.29 to 2.67	0.881
Pakistani/Bangladeshi	−0.5	−1.15 to 0.15	0.134	−0.49	−1.15 to 0.17	0.145	−0.2	−2.60 to 2.20	0.869	−0.15	−2.57 to 2.27	0.834
Others	−0.48	−1.10 to 0.15	0.134	−0.52	−1.15 to 0.11	0.108	0.52	−1.78 to 2.81	0.656	0.63	−1.67 to 2.92	0.592
	**HDL cholesterol**						**Total cholesterol**					
	**Waist to height ratio**			**Overweight status**			**Waist to height ratio**			**Overweight status**		
	**Coef**	**95% CI**	**p Value**	**Coef**	**95% CI**	**p Value**	**Coef**	**95% CI**	**p Value**	**Coef**	**95% CI**	**p Value**
Waist to height ratio, 11–13 years	−0.94	−1.87 to −0.02	0.045	–	–	–	0.73	−1.16 to 2.62	0.449	–	–	–
Overweight, 11–13 years (normal weight—Ref)												
Overweight/obese	–	–	–	−0.13	−0.23 to −0.02	0.017	–	–	–	−0.01	−0.32 to 0.38	0.906
Ethnicity (White UK—Ref)												
Black Caribbean	−0.01	−0.17 to 0.26	0.975	0.03	−0.13 to 0.20	0.699	0.05	−0.31 to 0.40	0.793	0.03	−0.34 to 0.36	0.965
Black African	0.05	−0.11 to 0.21	0.537	0.07	−0.08 to 0.23	0.335	−0.28	−0.62 to 0.05	0.095	−0.28	−0.62 to 0.05	0.1
Indian	−0.11	−0.29 to 0.06	0.207	−0.13	−0.30 to 0.04	0.142	−0.05	−0.43 to 0.32	0.787	−0.06	−0.43 to 0.31	0.752
Pakistani/Bangladeshi	0.04	−0.13 to 0.22	0.617	0.02	−0.15 to 0.19	0.857	0.05	−0.31 to 0.42	0.771	0.06	−0.30 to 0.42	0.746
Others	−0.03	−0.20 to 0.13	0.698	−0.05	−0.21 to 0.11	0.561	−0.06	−0.40 to 0.28	0.734	−0.05	−0.39 to 0.29	0.767

*Linear regression model with regression coefficients adjusted for ethnicity and waist to height ratio or overweight status at 11–13 years. Allostatic load score derived at 21–23 years using high-risk thresholds defined as below the 25th centile for high-density lipoprotein (HDL) cholesterol, and above the 75th centile for all other biomarkers. The thresholds were: systolic, 121.0 mm Hg; diastolic, 77 mm Hg; BMI, 26.8 kg/m^2^; waist to height ratio male, 0.89, and female, 0.86; total cholesterol, 4.8 mmol/L; HDL cholesterol, 1.8 mmol/L; glycated haemoglobin (HbA1c), 37.0 mmol/mol.

DASH, Determinants of young Adult Social well-being and Health.

## Discussion

This is the first UK study, as yet from a pilot follow-up, to report a prospective association between adiposity measures and cardiovascular disease (CVD) risk from early adolescence to early adulthood in an ethnically diverse cohort. At 11–13 years, 30% of the follow-up sample was overweight/obese compared with 39% at 21–23 years, with a larger increase between 14–16 years and 21–23 years than between 11–13 years and 14–16 years. There was a striking steady increase in smoking from early adolescence. Ethnic patterns in BP and vascular biomarkers varied by gender, and were unaltered after adjustment for adiposity or social factors. Of note was that from early adolescence, Black Caribbeans and Black Africans were more likely to be overweight than their White UK peers, with Black Caribbean females also having a higher HbA1c at 21–23 years. Regardless of ethnicity, adiposity measures were related to sBP at each age, and adiposity measures at 11–13 years were related to the AL, HbA1c and HDL at 21–23 years.

Growth acceleration for ethnic minorities from a young age may have had some impact. The Millennium Cohort Study showed accelerated growth from birth for ethnic minority children who were not preterm or small for dates.^18^ Black Caribbean babies were about 150 g lighter than their White British peers but by age 3 years they were about 1 kg heavier and 2 cm taller. Indian babies were about 290 g lighter at birth, showed a similar trend of growth acceleration such that by 5 years they were of similar weight and height. At ages <11 years, cross-sectional studies have shown that Black African-origin children are ∼3 cm taller and heavier and South Asians ∼2 cm shorter and lighter than Whites in Britain.^3^ ^19^ In DASH, the taller heights of the Black Caribbean and Black Africans at 11–13 years but not at 14–16 years^5^ or 21–23 years support accelerated growth patterning prior to 11–13 years. South Asians remained shorter at 21–23 years than White UK, a pattern consistent with other studies of young children.^19^ The larger body size for Black Caribbeans and Black Africans also correspond with other studies.^18^ ^20^

Childhood growth acceleration is associated with later obesity and CVD, via mechanisms including rises in BP, types of fat deposition as well as perhaps insulin resistance. Differential growth and metabolic factors between birth and 3 years of age between South Asians and Europeans suggest insulin levels change after those of adiposity, as also shown by adiponectin concentrations.^4^ That remains our hypothesis,^21^ not least because ‘insulin resistance’ fails to predict coronary heart disease (CHD), its excess or other vascular events in South Asians,^22^ or in other insulin resistance syndromes.^23^ Growth acceleration in infancy may be a critical period,^24^ as may be the ‘adiposity rebound’, thought to be around ages 5–8 years but often earlier in poorer, marginally or undernourished communities.^25^ Early studies in marginally nourished settings in Jamaica showed at ages 0–5 year that height and weight lagged 25% centile points behind Boston, USA, standards of the time.^26^ That study is probably representative of parents and their children who would be the grandparents and parents who migrated to Britain as the forebears of the Caribbean-origin sample here. Similarly, early child cohorts show the impact on child growth and early mortality, mostly from malaria, in West Africa. Over 50 years ago, infants, universally breast fed, gained weight and height similarly to the UK children for the first 6 months, then when weaned, growth faltered, although in the Gambian setting without overt malnutrition.^27^ Recent studies in Nigeria, with parasitaemia examined throughout pregnancy, suggest that birth dimensions were lower and infant BP by age 1 year had climbed faster, even adjusting for catch-up growth.^28^ Postnatal child growth is also affected by other inherent (eg, haemoglobin genotype) exposure to obesogenic neighbourhoods (eg, high density of fast food outlets),^29^ earlier maturation and mixed feeding as infants.

The ethnic-specific blood biomarker profiles suggest changing lipid profiles among Black Caribbeans. Earlier studies of adult migrant Caribbeans,^2^ ^30^ and those in the Caribbean,^31^ ^32^ showed lipid profiles that were protective of CHD—lower total, low-density lipoprotein (LDL) cholesterol and triglyceride concentrations and higher HDL cholesterol. Fasting bloods were not taken in DASH but total and HDL cholesterol, unaffected by fasting, were not different from White UK. This is consistent with previous reports of lipid profiles among 9–10 year-old^33^ and 35–44 year-old UK-born Black Caribbeans. The lower HDL cholesterol among South Asian males indicates some continuity of patterns for these groups. Contrary to what could be expected from the US-based studies and our own of 35–54-year-olds,^34^ ethnic differences in AL were not evident in early 20s. This raises questions about what might be mitigating biological ‘wear and tear’ in the context of disproportionate exposure to socioeconomic disadvantage and obesity from a younger age. Physical activity in the 20s (as measured by accelerometry) may have a protective effect.^35^

DASH contains a diverse sample, has high retention rates and low item-non-response, mainly due to enormous community support. The lack of data before age 11 years, and blood biomarkers only at 21–23 years, lack of body composition measures at age 11 years, and the small sample sizes for the ethnic groups in the pilot follow-up are limitations. Prior to DASH, a large-scale study with an explicit focus on ethnicity had not been attempted. Our pilot studies in 2001 suggested that response rates would be low if bloods were taken and starting at younger ages would incur a high loss to follow-up between primary and secondary schools. Measuring adiposity in ethnic minorities is not unproblematic but whether ethnic-specific bioelectrical impedance analysis calibration is required continues to be debated.^15^ ^19^ At 21–23 years, fat mass and lean mass were measured using Bodystat 1500MDD. The ethnic patterns in fat mass corresponded to those reported here using anthropometric measures. For example, among females the fat mass indices (kg/m^2^) were as follows—White UK 6.41 (95% CI 5.50 to 7.32), Black Caribbeans 9.13 (95% CI 7.80 to 10.46), Black Africans 8.01 (95% CI 7.02 to 9.00), Indians 7.71 (95% CI 6.67 to 8.74) and Pakistanis/Bangladeshis 7.43 (95% CI 6.43 to 8.42). The small sample size of the ethnic groups prohibited robust testing of ethnic-specific effects. The primary aims of the pilot follow-up were to locate the diverse groups in the cohort, investigate whether they would participate in a subsequent follow-up study and agree for their parents to be invited to join DASH, and whether they would consent to the different measures, notably the biomarkers. Missing data were low for anthropometry and BP but relatively high for the blood biomarkers which could have introduced bias. Despite these small numbers, these findings in a small age range provide a robust platform for planning future studies.

A recent stabilisation of obesity rates in children in England is good news but there is no room for complacency. Early adolescent overweight adversely affected CV health of 20-year-olds and overweight increased between late teens and early 20s, regardless of gender or ethnicity. Physical peak is expected in the 20s, a transitional age that is ignored in interventions. Urgent attention is required to prevent diminishing ‘peak’ health in a ‘coming of age’ generation that faces the additional challenges from economic precarity due to the economic recession.
