# Supplementary material for: Longitudinal study of cardiometabolic risk from early adolescence to early adulthood in an ethnically diverse cohort
Source: BMJ Open. 2016 Dec 15;6(12):e013221. doi: 10.1136/bmjopen-2016-013221 (PMC5223645; doi:10.1136/bmjopen-2016-013221)
Supplement: supplementary data [file bmjopen-2016-013221supp.pdf]

SUPPLEMENT MATERIAL

Supplement Table 1 Body size and blood pressure at 11-13y, 14-16y and 21-23y, and blood bio-markers at 21-23y, by ethnicity, age and gender in the DASH study

		Whites UK	Black Caribbean	Black African	Indian	Pakistani/Bangladeshi	Others
**Males**							
Height	11-13y	155.6 (153.8,157.4)	157.9 (156.1,159.8)	158.9 (157.2,160.6)*	151.0 (149.1,152.8)	152.5 (150.8,154.3)	154.9 (153.1,156.6)
	14-16y	172.5 (171.0,174.1)	171.9 (170.3,173.5)	172.7 (171.2,174.1)	166.2 (164.6,167.8)**	166.9 (165.4,168.4)**	170.8 (168.6,173.0)
	21-23y	179.1 (177.8,180.5)	177.7 (176.3,179.1)	178.4 (177.2,179.7)	173.0 (171.6,174.4)**	172.8 (171.5,174.2)**	175.4 (174.1,176.7)**
Weight	11-13y	47.6 (44.1,51.0)	53.8 (50.3,57.4)	54.0 (50.8,57.2)	45.3 (41.8,48.8)	44.9 (41.6,48.2)	51.5 (48.1,54.8)
	14-16y	58.4 (55.4,61.5)	65.9 (62.8,69.0)	65.4 (62.6,68.2)*	54.5 (51.4,57.6)	54.8 (51.9,57.8)	63.4 (59.1,67.6)
	21-23y	76.6 (73.5,79.7)	82.1 (78.9,85.3)	82.0 (79.1,84.9)	71.5 (68.3,74.8)	73.2 (70.2,76.2)	75.7 (72.6,78.7)
Waist	11-13y	66.1 (64.2,68.0)	67.5 (65.5,69.4)	64.6 (65.9,69.4)	65.6 (63.6,67.5)	64.9 (63.1,66.7)	66.4 (64.5,68.2)
	14-16y	72.6 (70.4,74.8)	74.9 (72.7,77.1)	75.2 (73.2,77.2)	70.1 (68.0,72.3)	71.0 (69.0,73.1)	74.7 (71.6,77.7)
	21-23y	83.9 (81.3,86.5)	84.9 (82.2,87.5)	85.3 (82.9,87.7)	83.9 (81.3,86.6)	84.4 (82.0,86.9)	83.2 (80.8,85.7)
Waist height ratio	11-13y	0.43 (0.41,0.44)	0.43 (0.42,0.44)	0.43 (0.41,0.44)	0.44 (0.42,0.45)	0.43 (0.41,0.44)	0.43 (0.42,0.44)
	14-16y	0.42 (0.41,0.43)	0.44 (0.42,0.45)	0.44 (0.42,0.45)	0.42 (0.41,0.43)	0.43 (0.41,0.44)	0.44 (0.42,0.45)
	21-23y	0.47 (0.45,0.48)	0.48 (0.46,0.49)	0.48 (0.46,0.49)	0.48 (0.47,0.50)	0.49 (0.47,0.50)	0.47 (0.46,0.49)
% Overweight/Obese	11-13y	16.7 (10.6,25.4)	39.4 (29.6,50.2)	37.6 (28.8,47.3)	25.3 (17.3,35.3)	18.0 (11.7,26.6)	31.0 (22.6,40.8)*
	14-16y	10.3 (5.8,17.7)	30.4 (22.2,40.0)	31.1 (23.7,39.5)*	17.3 (11.0,16.2)	13.5 (8.3,21.3)	11.3 (6.6,18.5)
	21-23y	35.2 (26.1,45.6)	53.0 (42.5,63.3)	47.0 (37.6,56.6)	38.1 (28.4,48.8)	38.3 (29.2,48.3)	36.7 (27.7,46.8)
Systolic blood pressure	11-13y	110.4 (108.4,112.4)	109.7 (107.6,111.7)	110.3 (108.4,112.1)	108.7 (106.6,110.7)	107.4 (105.4,109.3)	108.7 (106.7,110.6)
	14-16y	116.1 (114.0,118.2)	117.1 (114.9,119.2)	117.5 (115.6,119.5)	113.9 (111.8,116.0)	114.0 (112.0,116.0)*	114.5 (111.5,117.4)
	21-23y	112.5 (120.6,124.5)	121.4 (119.4,123.4)	121.3 (119.5,123.2)	118.9 (116.9,120.9)**	117.3 (115.4,119.2)***	119.2 (117.4,121.1)**
HbA1c, mmol/mol	21-23y	34.1 (33.03,35.2)	36.0 (34.9,37.2)	35.0 (33.9,36.0)	35.0 (33.9,36.1)	34.4 (33.4,35.5)	34.5 (33.5,35.6)
HDL Cholesterol, mmol/l	21-23y	1.48 (1.40,1.56)	1.52 (1.43,1.60)	1.50 (1.42,1.58)	1.33 (1.25,1.42)*	1.40 (1.31,1.48)*	1.46 (1.38,1.54)
Total Cholesterol, mmol/l	21-23y	4.28 (4.10,4.47)	4.29 (4.09,4.48)	4.02 (3.84,4.20)*	4.31 (4.12,5.41)	4.51 (4.33,4.69)*	4.27 (4.10,4.45)
Allostatic Load	21-23y	2.92 (2.55,3.29)	2.96 (2.57,3.34)	2.63 (2.25,3.01)	2.69 (2.31,3.08)	2.63 (2.26,3.01)	2.49 (2.13,2.84)
**Females**							
Height	11-13y	155.9 (154.1,157.6)	158.2 (156.3,160.0)	159.2 (157.6,160.8)	151.2 (149.3,153.1)***	152.8 (151.0,154.8)**	155.1 (153.4,156.9)
	14-16y	164.1 (162.5,165.7)	163.4 (161.8,164.9)	164.2 (162.8,165.5)	157.7 (156.1,159.3)***	158.4 (156.8,160.0)***	162.3 (160.2,164.5)*
	21-23y	166.0 (164.6,167.3)	164.5 (163.1,165.9)	165.3 (164.1,166.5)	159.8 (158.4,161.3)***	159.7 (158.3,161.1)***	162.3 (160.9,163.6)**
Weight	11-13y	48.8 (45.4,52.2)	55.0 (51.5,58.5)***	55.2 (52.1,58.3)**	46.5 (42.9,50.1)	46.1 (42.7,49.6)	52.7 (49.3,56.0)
	14-16y	54.5 (51.4,57.6)	62.0 (59.0,65.0)***	61.5 (58.9,64.2)**	50.6 (47.4,53.7)	50.9 (47.9,54.0)	59.5 (55.3,63.6)
	21-23y	65.7 (62.6,68.8)	71.1 (68.0,74.3)**	71.1 (68.3,73.9)*	60.6 (57.3,63.9)	62.2 (59.1,65.3)	64.7 (61.7,67.7)
Waist	11-13y	66.3 (64.4,68.2)	67.7 (65.7,69.6)*	67.8 (66.1,69.5)*	65.8 (63.8,67.8)	65.1 (63.2,67.0)	66.6 (64.7,68.4)
	14-16y	70.2 (68.0,72.4)	72.5 (70.3,74.6)*	72.8 (70.9,74.6)*	67.7 (65.5,70.0)	68.6 (66.4,70.8)	72.3 (69.3,75.2)
	21-23y	79.8 (77.3,82.4)	80.8 (78.2,83.4)*	81.3 (78.9,83.7)	79.9 (77.2,82.6)	80.4 (77.9,83.0)	79.2 (76.7,81.7)

Waist height ratio	11-13y	0.43 (0.41,0.44)	0.43 (0.42,0.44)*	0.43 (0.42,0.44)	0.45 (0.42,0.45)	0.43 (0.41,0.44)	0.43 (0.42,0.44)
	14-16y	0.43 (0.42,0.44)	0.44 (0.43,0.46)**	0.44 (0.43,0.45)*	0.43 (0.42,0.44)	0.43 (0.42,0.45)	0.44 (0.43,0.46)
	21-23y	0.48 (0.47,0.50)	0.49 (0.48,0.51)**	0.49 (0.48,0.51)	0.50 (0.48,0.51)*	0.50 (0.49,0.52)	0.49 (0.47,0.50)
% Overweight/Obese	11-13y	18.7 (12.0,27.9)	42.7 (32.8,53.2)***	40.8 (32.1,50.2)**	27.9 (19.3,38.6)	20.1 (13.1,29.5)	33.9 (25.1,44.0)
	14-16y	12.8 (7.0,22.1)	33.8 (24.3,44.8)**	34.2 (25.6,44.0)**	19.9 (12.3,30.5)	16.2 (9.7,26.0)	29.3 (17.6,44.7)*
	21-23y	30.3 (22.0,40.2)	47.4 (37.3,57.7)**	41.5 (32.9,50.7)*	33.0 (23.9,43.5)	33.2 (24.4,43.2)	31.7 (23.4,41.4)
Systolic blood pressure	11-13y	108.3 (106.3,110.3)	107.6 (105.6,109.6)	108.2 (106.4,110.0)	106.6 (104.5,108.7)	105.3 (103.3,107.3)	106.6 (104.7,108.5)
	14-16y	106.8 (104.6,108.9)	107.8 (105.7,109.8)	108.2 (106.4,110.0)	104.6 (102.4,106.8)	104.7 (102.5,106.8)	105.1 (102.2,108.0)
	21-23y	109.7 (107.8,111.7)	108.6 (106.6,110.5)	108.5 (106.8,110.3)	106.1 (104.1,108.2)	104.5 (102.6,106.5)*	106.4 (104.6,108.3)
HbA1c, mmol/mol	21-23y	33.5 (32.4,34.6)	35.4 (34.3,36.5)*	34.3 (33.3,35.4)	34.4 (33.2,35.5)	33.8 (32.7,34.9)	33.9 (32.8,34.9)
HDL Cholesterol, mmol/l	21-23y	1.65 (1.57,1.73)	1.69 (1.60,1.77)	1.67 (1.59,1.75)	1.50 (1.42,1.59)	1.57 (1.48,1.65)	1.64 (1.56,1.72)
Total Cholesterol, mmol/l	21-23y	4.39 (4.20,4.57)	4.39 (4.20,4.57)	4.12 (3.94,4.29)	4.42 (4.22,4.61)	4.61 (4.42,4.80)	4.38 (4.20,4.55)
Allostatic Load	21-23y	2.29 (1.91,2.66)	2.32 (1.94,2.70)	2.00 (1.62,2.37)	2.06 (1.66,2.46)	2.00 (1.61,2.38)	1.85 (1.49,2.21)

*p<0.05, **p<0.01, ***p<0.001. P-values were derived from simple linear/logistic (overweight) regression models with ethnicity as an independent variable.

Supplement Table 2: Diastolic blood pressure from adolescence to early adulthood for males and females in the DASH study: association with longitudinal measures of adiposity*

Covariates	Waist to height ratio			Overweight Status		
	Coef	95% CI	p-value	Coef	95% CI	p-value
**Males**						
**Waist to height ratio**	0.32	(20.8,43.6)	<0.001	-	-	-
**Overweight (Normal weight - Ref)**						
Overweight/Obese	-	-	-	2.91	(0.36,4.02)	0.019
**Age (11-13y - Ref)**						
14-16y	49.5	(48.2,50.9)	<0.001	49.5	(49.1,50.9)	<0.001
21-23y	51.7	(50.3,53.2)	<0.001	53.8	(52.3,55.3)	<0.001
**Ethnicity (White UK - Ref)**						
Black Caribbean	-0.76	(-3.54,2.01)	0.589	-1.32	(-4.16,1.51)	0.359
Black African	-0.90	(-3.53,1.74)	0.504	-1.36	(-4.06,1.34)	0.324
Indian	-2.14	(-4.84,0.56)	0.121	-2.26	(-5.02,0.49)	0.107
Pakistani/Bangladeshi	-4.54	(-7.14,-1.93)	0.001	-4.34	(-7.0,-1.68)	0.001
Others	-4.01	(-6.72,-1.31)	0.004	-4.14	(-6.91,1.37)	0.003
**Employment**						
No	0.33	(-1.25,1.92)	0.680	0.23	(-1.35, 1.81)	0.776
Not stated	0.98	(-1.3,3.27)	0.401	0.62	(-1.65,2.89)	0.594
**Smoking**						
**Yes**	0.90	(-0.6,2.41)	0.238	0.96	(-0.55,2.47)	0.215
Not stated	2.09	(0.33,3.85)	0.020	2.30	(0.54,4.05)	0.011
**Females**						
**Waist to height ratio**	28.7	(20.2,37.1)	<0.001	-	-	-
**Overweight (Normal weight - Ref)**						
Overweight/Obese	-	-	-	3.09	(1.70,4.48)	<0.001
**Age (11-13y - Ref)**						
14-16y	39.5	(38.4,40.6)	<0.001	39.9	(38.8,41.0)	<0.001
21-23y	39.1	(37.8,40.3)	<0.001	41.9	(40.7,43.1)	<0.001
**Ethnicity (White UK - Ref)**						
Black Caribbean	0.37	(-1.73,2.47)	0.728	0.72	(-1.43,2.87)	0.511
Black African	0.63	(-1.36,2.62)	0.536	0.71	(-1.32,2.73)	0.495
Indian	-1.51	(-3.72,0.71)	0.182	-1.19	(-3.44,1.06)	0.301
Pakistani/Bangladeshi	-0.82	(-3.0,1.35)	0.457	-0.50	(-2.72,1.72)	0.658
Others	-1.03	(-3.1,1.1)	0.343	-0.95	(-3.12,1.22)	0.39
**Employment**						
No	-0.44	(-1.64,0.76)	0.469	-0.44	(-1.65,0.75)	0.467

	Not stated	0.61	(-1.57,2.78)	0.581	0.38	(-1.74,2.5)	0.725
**Smoking**							
	Yes	0.46	(-0.78,1.71)	0.467	0.57	(-0.68,1.81)	0.372
	Not stated	0.31	(-1.02,1.65)	0.648	0.06	(-1.27, 1.39)	0.931

*Mixed-Effects Linear Regression Model with regression coefficients adjusted for age, ethnicity, waist to height ratio or overweight status, parental (11-13y and 14-16y)/own (21-23y) employment and currently Smoking

Supplement Table 3: The influence of change in waist to height ratio or overweight status on HbA1c at 21-23y in the DASH study

	**HbA1c***					
	**Waist to height ratio**			**Overweight Status**		
	**Coef**	**95% CI**	**p-value**	**Coef**	**95% CI**	**p-value**
**Males**						
**Waist to height ratio change between 11-13y and 21-23y**	-1.19	(-10.3, 7.9)	0.797	-	-	-
**Weight change (Normal weight at 11-13y-normal weight at 21-23y)**						
Normal weight at 11-13y-Overweight/Obese at 21-23y	-	-	-	-0.77	(-2.05,0.51)	0.24
Overweight/Obese at 11-13y to Normal weight at 21-23y	-	-	-	0.67	(-1.46, 2.8)	0.535
Overweight/Obese at 11-13y to Overweight/Obese at 21-23y	-	-	-	0.89	(-0.34,2.13)	0.157
**Ethnicity (White UK - Ref)**						
Black Caribbean	0.93	(-0.86,2.72)	0.309	0.7	(-1.06,2.45)	0.435
Black African	0.71	(-1.04,2.47)	0.426	0.79	(-0.9,2.48)	0.361
Indian	1.26	(-0.46,2.98)	0.152	1.34	(-0.34,3.02)	0.118
Pakistani/Bangladeshi	0.65	(-1.01,2.32)	0.438	0.57	(-1.03,2.18)	0.481
Others	0.52	(-1.78,2.81)	0.863	-0.04	(-1.69,1.61)	0.962
**Females**						
**Waist to height ratio change between 11-13y and 21-23y**	-3.04	(-14.3, 8.26)	0.596	-	-	-
**Weight change (Normal weight at 11-13y-normal weight at 21-23y)**						
Normal weight at 11-13y-Overweight/Obese at 21-23y	-	-	-	0.43	(-1.72,2.59)	0.691
Overweight/Obese at 11-13y to Normal weight at 21-23y	-	-	-	3.62	(0.95,6.29)	0.008
Overweight/Obese at 11-13y to Overweight/Obese at 21-23y	-	-	-	1.14	(-0.58,2.86)	0.193
**Ethnicity (White UK - Ref)**						
Black Caribbean						
Black African	3.23	(0.73,5.72)	0.011	2.58	(0.23,4.93)	0.031
Indian	0.97	(-1.48,3.41)	0.426	0.46	(-1.8,2.72)	0.688
Pakistani/Bangladeshi	0.46	(-2.22,3.15)	0.152	0.38	(-2.11,2.87)	0.763
Others	0.00	(-2.54,2.54)	1.000	-0.29	(-2.72,2.15)	0.817
	0.79	(-1.65,3.23)	0.524	0.75	(-1.55,3.06)	0.521

*Mixed-Effects Linear Regression Model with regression coefficients adjusted for age, ethnicity, waist to height ratio or overweight status

Supplement Table 4: The influence of change in waist to height ratio or overweight status on HDL and Total Cholesterol at 21-23y in the DASH study

**Males**												
	**HDL Cholesterol***						**Total Cholesterol***					
	**Waist to height ratio**			**Overweight Status**			**Waist to height ratio**			**Overweight Status**		
**Covariates**	**Coef**	**95% CI**	**p-value**	**Coef**	**95% CI**	**p-value**	**Coef**	**95% CI**	**p-value**	**Coef**	**95% CI**	**p-value**
**Waist to height ratio change between 11-13y and 21-23y**	-1.59	(-2.29,-0.89)	<0.001	-	-	-	3.26	(1.46,5.05)	<0.001	-	-	-
**Weight change (Normal weight at 11-13y-normal weight at 21-23y)**												
Normal weight at 11-13y-Overweight/Obese at 21-23y	-	-	-	-0.05	(-14.8,0.05)	0.367	-	-	-	0.54	(0.29,0.79)	<0.001
Overweight/Obese at 11-13y to Normal weight at 21-23y	-	-	-	0.29	(0.13,0.46)	0.001	-	-	-	-0.06	(-0.49,0.36)	0.78
Overweight/Obese at 11-13y to Overweight/Obese at 21-23y	-	-	-	-0.13	(-0.22,-0.03)	0.009	-	-	-	0.19	(-0.05,0.44)	0.119
**Ethnicity (White UK - Ref)**												
Black Caribbean	0.14	(-0.01,27.7)	0.975	0.08	(-0.05,0.22)	0.238	-0.03	(-0.38,0.32)	0.793	-0.08	(-0.42,0.26)	0.646
Black African	0.05	(-0.09,0.19)	0.469	-0.02	(-0.15,0.11)	0.782	-0.33	(-0.68,0.02)	0.062	-0.23	(-0.56,0.11)	0.181
Indian	-0.13	(-0.26,0.02)	0.054	-0.18	(-0.31,-0.05)	0.006	0.20	(-0.14,0.55)	0.247	0.14	(-0.19,0.47)	0.402
Pakistani/Bangladeshi	-0.12	(-0.6,0.01)	0.063	-0.18	(-0.31,-0.05)	0.006	0.29	(-0.04,0.62)	0.089	0.35	(0.03,0.67)	0.031
Others	-0.04	(-0.09,16.9)	0.539	-0.03	(-0.16,0.10)	0.615	0.05	(-0.28,0.38)	0.778	0.05	(-0.28,0.38)	0.753
**Females**												
**Waist to height ratio change between 11-13y and 21-23y**	-1.15	(-1.92,-0.38)	0.003	-	-	-	2.82	(1.22,4.43)	0.001	-	-	-
**Overweight, 11-13y (Normal weight - Ref)**												
Normal weight at 11-13y-Overweight/Obese at 21-23y	-	-	-	-0.08	(-0.23,0.06)	0.261	-	-	-	0.38	(0.07,0.69)	0.018
Overweight/Obese at 11-13y to Normal weight at 21-23y	-	-	-	0.09	(-0.10,27.4)	0.341	-	-	-	-0.12	(-0.51,0.27)	0.562
Overweight/Obese at 11-13y to Overweight/Obese at 21-23y	-	-	-	-0.22	(-0.34,-0.10)	<0.001	-	-	-	0.15	(-0.10, 0.40)	0.233
**Ethnicity (White UK - Ref)**												
Black Caribbean	0.05	(-0.12,0.22)	0.553	0.04	(-0.12,0.21)	0.605	-0.01	(-0.36,0.35)	0.989	0.01	(-0.34,0.36)	0.965
Black African	0.07	(-0.10,0.23)	0.42	0.08	(-0.07,0.24)	0.335	-0.37	(-0.71,-0.02)	0.034	-0.30	(-0.64,0.03)	0.074
Indian	-0.09	(-0.27,0.09)	0.31	-0.12	(-0.29,0.05)	0.182	-0.17	(-0.55,0.20)	0.367	-0.07	(-0.44,0.29)	0.697
Pakistani/Bangladeshi	0.05	(-0.12,0.23)	0.569	0.01	(-0.16,0.19)	0.863	0.03	(-0.33,0.39)	0.881	0.11	(-0.25,0.47)	0.558
Others	-0.02	(-0.29,0.14)	0.786	-0.03	(-0.19,0.13)	0.72	-0.10	(-0.45,0.24)	0.055	-0.02	(-0.36,0.32)	0.907

* Linear Regression Model with regression coefficients adjusted for ethnicity and waist to height ratio or overweight status at 11-13y
